# Effect of Myo-Inositol Supplementation in Polycystic Ovary Syndrome—Scoping Review

**DOI:** 10.3390/nu18132093

**Published:** 2026-06-26

**Authors:** Julia Habryka, Maja Ławniczek, Sabina Krupa-Nurcek

**Affiliations:** 1 Faculty of Medicine, Collegium Medicum, University of Rzeszów, 35-310 Rzeszów, Poland; julhab99@gmail.com (J.H.); maajanka@gmail.com (M.Ł.); 2 Department of Surgery, Faculty of Medicine, Collegium Medicum, University of Rzeszów, 35-310 Rzeszów, Poland

**Keywords:** myo-inositol, polycystic ovary syndrome, PCOS, ovarian function, ART, ovulation

## Abstract

**Objectives**: Polycystic ovary syndrome (PCOS) is a widespread and complex endocrine disorder affecting women of childbearing potential, characterized by reproductive dysfunction, hyperandrogenism, and metabolic disorders, including insulin resistance. Insulin resistance is a key pathogenetic factor contributing to ovarian dysfunction and reduced fertility. Myo-inositol (MI), a ubiquitous polyol, has earned a reputation as a promising dietary supplement due to its vital role in insulin signaling pathways. This scoping review aimed to map the available scientific literature on the effects of MI supplementation in women with PCOS, with particular emphasis on fertility and ovarian function, and to identify gaps in the current evidence base. **Methods**: This scoping review was conducted in accordance with the methodology developed by the Joanna Briggs Institute (JBI) and presented in accordance with the Preferred Reporting Items for Systematic Reviews and Meta-Analyses for Scoping Reviews (PRISMA-ScR) guidelines. A literature search was conducted against six electronic databases: PubMed, Scopus, Web of Science, EBSCO (MEDLINE Complete), Cochrane Library and Google Scholar. Searches were conducted between 10 January and 20 February 2026. Eligibility sources included original articles (observational and randomized controlled trials), meta-analyses, systematic and narrative reviews, published in English with full text available, focusing on adult women with PCOS. Data extraction was performed independently by two reviewers using the Population–Concept–Context (PCC) framework. In accordance with the scope review methodology, no formal critical appraisal of study quality and no quantitative synthesis were performed. This is consistent with JBI methodology, which does not require critical appraisal for scoping reviews unless explicitly justified. **Results**: Of the 77 records initially identified, 13 studies were included in the review, and no duplicates were found. These potential benefits should be interpreted cautiously, as the available evidence is heterogeneous and varies across study designs. Potential benefits were also reported for hormonal and metabolic parameters, including reductions in hyperandrogenism and the improvement of insulin sensitivity. Some studies suggest benefits for oocyte and embryo quality, but results remain inconsistent. **Conclusions**: MI supplementation may support PCOS management, particularly in fertility-related outcomes. Its ability to improve ovulation, increase pregnancy rates, optimize ART outcomes, and mitigate the risk of OHSS highlights its clinical utility. However, the evidence remains heterogeneous, and some outcomes, particularly oocyte and embryo quality, remain inconclusive.

## 1. Introduction

**Figure 1:**
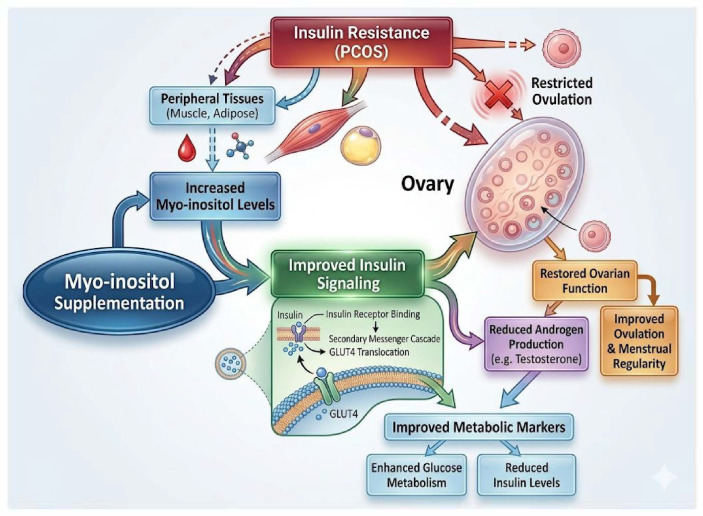
Mechanism of action of MI supplementation in patients suffering from PCOS. Key molecular events include the restoration of insulin receptor signaling and GLUT4 translocation, adapted from established physiological frameworks [7,8,10,11] (AI-generated).

PCOS is a complex endocrine disorder affecting a woman’s reproductive age and characterized by irregular menstrual cycles, clinical or biochemical hyperandrogenism, and polycystic ovary morphology seen on ultrasound examination [1,2]. It is estimated that the prevalence of PCOS in the general population of women ranges from 5% to 15%, depending on the diagnostic criteria used [3,4]. PCOS is a heterogeneous condition that is associated not only with reproductive problems such as infertility, but also with an increased risk of developing metabolic disorders, including insulin resistance, type 2 diabetes, dyslipidemia, obesity, and cardiovascular disease [5,6]. Insulin resistance is considered a key pathogenetic factor in PCOS, contributing to hyperandrogenism and ovulatory dysfunction [7]. In addition to standard pharmacological treatment methods and lifestyle modifications, dietary supplements are gaining more and more interest. Among them, MI, one of the nine isomers of inositol, has attracted particular interest because of its role in intracellular insulin signaling pathways [8,9]. MI is a precursor of secondary inositol phosphate messengers, which play a key role in insulin signal transduction, affecting glucose uptake and lipid metabolism [10]. It is believed that women with insulin resistance, including PCOS, are deficient or impaired in inositol metabolism, which can lead to impaired insulin action and worsening of symptoms [11]. In recent years, numerous studies have been conducted to evaluate the impact of MI supplementation on various aspects of PCOS, such as hormonal profile, metabolic parameters, regularity of menstrual cycles, oocyte quality, and fertility rates [12,13]. While the results of many studies are promising, the existence of differences in supplementation protocols, doses, duration of interventions, and heterogeneity of the populations of women with PCOS studied may lead to heterogeneous conclusions. However, the strength of evidence differs between outcomes, and findings reported in the literature are not always consistent. As a result, the reported findings are not uniform across all outcomes, and some potential benefits appear to be better supported than others. Figure 1 shows the mechanism of action of MI supplementation on patients suffering from PCOS.

The aim of this scoping review was to map and summarize the available scientific literature on the effect of MI supplementation on PCOS. This review aims to identify key research areas, types of interventions and outcomes most frequently reported, as well as to identify possible gaps in current evidence, which can serve as a basis for further clinical trials and the development of therapeutic guidelines.

## 2. Materials and Methods

### 2.1. Study Design

This investigation was structured as a scoping review because its aim was to chart the scope and characteristics of existing research on the effects of MI supplementation in women diagnosed with PCOS, with particular emphasis on fertility-related and ovarian outcomes. This methodological approach was selected due to the broad, multifaceted, and often heterogeneous nature of the literature concerning MI’s influence on different dimensions of PCOS, which makes it difficult to synthesize using a traditional systematic review format. A scoping review was therefore chosen to provide an overview of the evidence landscape, highlight central concepts, and identify areas where research remains limited, rather than to appraise study quality or calculate pooled effect estimates [14].

Our review was prepared following the methodology developed by the JBI and reported in accordance with the PRISMA-ScR guidelines [15,16]. This review included studies evaluating MI alone as well as MI in combination with other compounds (e.g., D-chiro-inositol, alpha-lipoic acid, melatonin), and these formulations were charted separately to ensure clarity regarding the type of intervention assessed.

### 2.2. Inclusion and Exclusion Criteria

We formulated a research question that explicitly outlined the PCC framework guiding this review. This allowed us to pinpoint the essential dimensions of MI supplementation in the context of PCOS and to systematically map the outcomes and associated risk factors reported in the literature [17].

The inclusion criteria encompassed: all peer-reviewed publications; original research articles (both observational studies and randomized controlled trials); meta-analyses; systematic reviews; narrative reviews; studies available in full text; papers written in English; research involving human participants; and studies published within the last decade (2016–2026).

The exclusion criteria comprised the following: case reports; commentaries; letters to the editor; book chapters; publications without accessible full text; articles written in languages other than English; studies conducted on animals; and research published outside the defined 10-year window (2016–2026).

**Population (P)**: The review included studies describing the effects of MI supplementation in women diagnosed with PCOS. Studies focusing on adult women were primarily considered, although some broader reviews encompassing reproductive age were also included if relevant to the overarching theme.

**Concept (C)**: The focus was on the impact of MI supplementation, including its various formulations (e.g., combination with D-chiro-inositol (DCI), alpha-lipoic acid (ALA), melatonin), on different outcomes related to PCOS. These outcomes included, but were not limited to, hormonal profile (e.g., androgens), metabolic parameters (e.g., insulin sensitivity, glycemic control, lipid profile, body mass index (BMI), ovarian function (e.g., ovulation, oocyte quality), reproductive outcomes (e.g., pregnancy rates, ART outcomes), and management of PCOS symptoms.

**Context (C)**: The studies included in this review were conducted within the clinical and research context of PCOS management. This encompassed various healthcare settings, geographical locations, and diverse interventional approaches to MI supplementation. The context also considered different study designs, from randomized controlled trials to reviews and in silico studies, to provide a comprehensive overview of the evidence.

*Types of Studies*: The review covered a wide range of study types, including original research (RCTs, observational studies), systematic reviews, meta-analyses, and narrative reviews, to ensure broad coverage of the existing evidence base.

### 2.3. Search Strategy

A broad search of the literature was performed across six major electronic sources: PubMed, Scopus, Web of Science, EBSCO (MEDLINE Complete), the Cochrane Library, and Google Scholar. The search encompassed studies available up to early 2026 and was carried out between 10 January and 20 February 2026, in accordance with the predefined protocol. The search methodology was developed following PRISMA-ScR and JBI recommendations to ensure clarity, transparency, and reproducibility [15,16]. PRISMA checklist is avaliable in Supplementary Materials.

The search strategy integrated both controlled indexing terms (such as MeSH headings in PubMed) and free-text expressions related to MI, PCOS, and their associated outcomes. Additional filters were applied to limit the results to human studies, English-language publications, and articles with accessible full texts. Eligible study designs included observational research, randomized controlled trials, systematic reviews, narrative reviews, and meta-analyses. The complete PubMed search string is provided as an example of the standardized and reproducible approach used across all databases.

For Scopus, Web of Science, EBSCO, the Cochrane Library, and Google Scholar, the search syntax was adjusted to match the indexing systems of each platform. Boolean operators (AND/OR) and controlled vocabulary (when available) were used to ensure comprehensive retrieval of relevant literature. The central search themes were consistent across all databases:

Concept 1: Myo-inositol supplementation (e.g., “myo-inositol,” “inositol,” “D-chiro-inositol,” “inositol supplementation”).

Concept 2: Polycystic Ovary Syndrome (e.g., “PCOS,” “polycystic ovarian syndrome”).

All identified records, screening outcomes, and reasons for exclusion will be documented in a PRISMA-ScR flow diagram. A summary of the search strategy is presented in Table 1.

### 2.4. Data Extraction

A standardized data extraction form, prepared in accordance with JBI guidelines for scoping reviews [15] and adapted to the specificities of this review, was used to compile key information from the analyzed publications. The data extraction process—referred to as “data charting” in scoping reviews [14,15]—was carried out independently by two reviewers. The PCC scheme was used to guide the identification of relevant information within the selected studies.

The form included: bibliographic data (e.g., Author, year, country), study aim, participant characteristics (e.g., number of participants, diagnosis), type of research (e.g., Randomized Controlled Trial, Review, Meta-analysis, retrospective case–control, in silico study), specific MI formulation and dosage, intervention duration, primary and secondary outcomes evaluated, and a summary of key results and findings (as exemplified in Table 2).

Before undertaking the full extraction phase, the reviewers first tested the data-collection form on a small group of randomly chosen studies to confirm that all required information could be captured clearly and without ambiguity. At this stage, the form was modified wherever the pilot work revealed gaps or unclear elements. The subsequent extraction was performed independently by two reviewers who had previously aligned their procedures by jointly extracting information from three preliminary articles. This preparatory step was intended to harmonize their understanding of the form’s categories and reduce the likelihood of inconsistent coding. Once sufficient agreement had been reached, they proceeded with the complete extraction process. Any disagreements between the reviewers were addressed through discussion until a shared conclusion was reached, and a third reviewer was involved only when consensus could not be achieved. In line with established scoping review practices, no formal inter-rater reliability statistics (such as a kappa coefficient) were calculated, as the aim of the review was to map the existing evidence base rather than to evaluate the methodological rigor of the included studies.

All study types were extracted into a single table to provide a comprehensive overview of the evidence landscape, consistent with the purpose of a scoping review. The table is descriptive and does not imply comparability or hierarchy between study designs.

### 2.5. Critical Appraisal Process

A scoping review may include a review of current evidence without including a methodological assessment of the included studies [15]. Therefore, formal critical appraisal of the methodological quality of the included studies was not explicitly conducted, consistent with the methodology of scoping reviews, which focuses on mapping and summarizing available evidence.

### 2.6. Process for Including Publications in the Review

**Figure 2:**
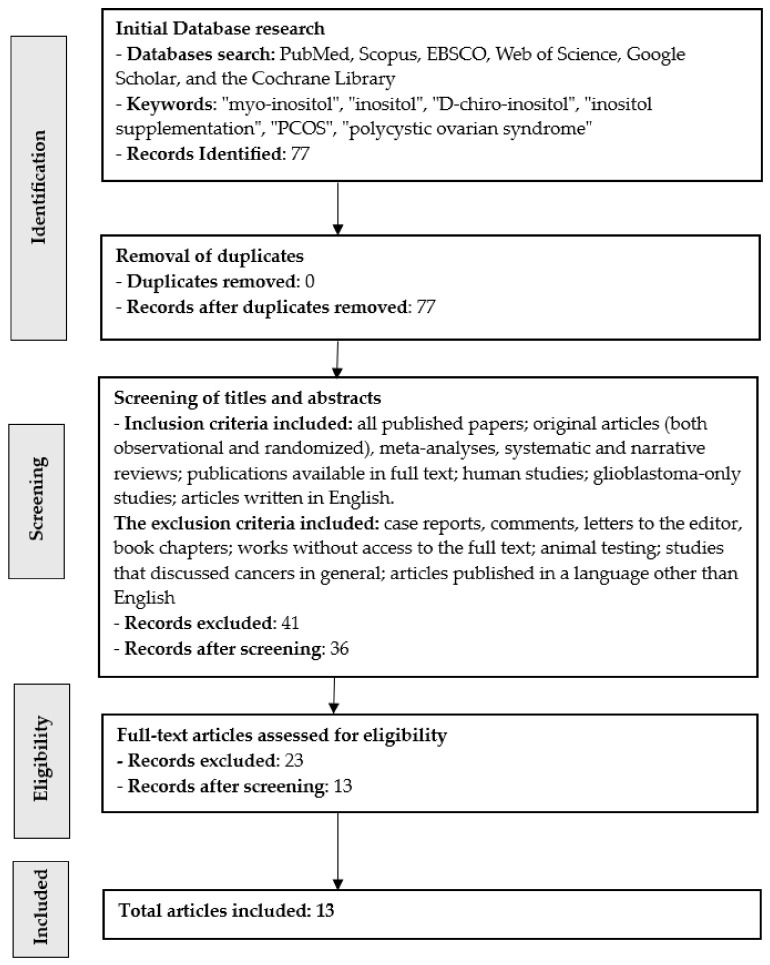
Literature search and selection flowchart for this review.

Our scoping review initially identified a total of 77 articles and ultimately included 13 articles in the final analysis (Figure 2) that examined the effect of MI supplementation in women with PCOS. There were no duplicate articles. After reviewing titles and abstracts according to the inclusion and exclusion criteria (*n* = 41), 36 articles remained for full-text screening. Twenty-three publications did not provide access to the full text and were excluded. As a result, after meeting all requirements, 13 publications were included in the review. The studies were conducted in various countries, including the USA (*n* = 2), Turkey (*n* = 1), Kosovo (*n* = 1), Taiwan (*n* = 1), Korea (*n* = 2), Germany (*n* = 2), Belgium (*n* = 1), Japan (*n* = 1), and Sweden (*n* = 1), reflecting a wide geographic range (Table 2).

### 2.7. Selection Process

The study selection procedure followed the PRISMA-ScR framework to maintain methodological clarity and reduce the likelihood of biased inclusion. All citations identified through database searches were transferred into the Zotero reference management system. At every stage of screening, two reviewers worked independently. The process began with a separate assessment of titles and abstracts, after which full texts of potentially relevant studies were examined. Each reviewer made decisions autonomously, and any inconsistencies between their judgments were addressed through discussion until a mutual agreement was reached. When additional input was required, a third reviewer provided an external opinion. In alignment with JBI recommendations for scoping reviews, no statistical measures of inter-reviewer reliability (such as a kappa statistic) were computed, as the objective of the review was to map existing evidence rather than evaluate the methodological rigor of included studies. The entire selection workflow is depicted in a PRISMA-ScR-compliant flow diagram (Figure 1). The selection procedure adhered to the PRISMA-ScR structure and consisted of four sequential phases:

Identification: All records retrieved from the designated databases (PubMed, Scopus, Web of Science, EBSCO, Cochrane Library, Google Scholar) were uploaded into a reference management tool.

Screening (Initial Filtering): Two reviewers independently examined titles and abstracts using predefined eligibility criteria. Studies that clearly failed to meet these criteria were removed during this stage.

Eligibility (Full-Text Assessment): Full texts of studies that passed the screening phase were reviewed independently by both reviewers. Any disagreements regarding study eligibility were resolved through discussion and consensus, with the option of consulting a third reviewer if needed.

Inclusion: Studies that fulfilled all PCC requirements were ultimately incorporated into the review. The number of records retained at each stage is presented in a PRISMA-ScR-compliant flow diagram (Figure 1).

### 2.8. Selection of Sources of Evidence Section

Two independent reviewers carried out the selection at each stage, ensuring independence in the process. Discrepancies were resolved through discussion and consensus, with a third reviewer consulted if necessary. The entire selection process was meticulously documented in accordance with PRISMA-ScR and JBI guidelines to ensure transparency and reproducibility [15,16,17].

## 3. Key Findings on the Effects of MI Supplementation in PCOS

PCOS is one of the leading causes of anovulatory infertility in women of reproductive age, manifested, among other things, by irregular ovulation or lack thereof, which is a consequence of ovarian dysfunction [1,2]. These disorders are often associated with insulin resistance and hyperandrogenism, which negatively affect the development of ovarian follicles and oocyte quality [3,4]. In this context, MI supplementation has been investigated as a potential supportive intervention foe improving selected fertility- and ovarian-related outcomes in women with PCOS.

### 3.1. Improving Ovulatory Function and Regularity of Menstrual Cycles

Among the outcomes most frequently reported in the available literature are menstrual regularity and ovulatory function. Several included studies suggested that MI supplementation may be associated with improvement in these parameters in some women with PCOS. MI may improve ovarian insulin sensitivity, which supports proper follicle maturation [31]. Insulin resistance increases ovarian androgen production, which disrupts follicle development and ovulation [32]. By reducing insulin resistance, MI can help to lower androgen levels and restore the delicate hormonal balance necessary for ovulation. It is important to note that these mechanisms are proposed based on selected studies, and not all research consistently confirms these effects. A meta-analysis conducted by Unfer et al., taking into account many clinical trials, reported improved ovulation rates and menstrual cycle regularity among women with PCOS receiving MI supplementation [12]. However, other analyses have reported no statistically significant effects, indicating that findings across studies remain inconsistent. The data reveal a significant risk of insulin resistance in obese individuals who suffer from PCOS [33]. Ciotta et al. also noted that MI may be useful in the treatment of PCOS patients undergoing ovulation induction, both for its insulin-sensitizing activity, and its role in oocyte maturation [34]. Regidor et al. reported improved cycle regularity and ovulation after several months of MI supplementation [35]. Although these findings are encouraging, other analyses report no statistically significant effects, underscoring the variability and inconsistency of the current evidence base.

### 3.2. Pregnancy-Related Outcomes and Findings in ART

Some studies report potential benefits of MI, but differences in study design and outcomes limit firm conclusions. In this context, MI supplementation has been investigated as a supportive intervention, with some studies suggesting potential benefits for selected ART-related and pregnancy-related outcomes. In the Sene AA review. et al., MI or its combination with DCI, was associated with improved clinical pregnancy rates in women with PCOS undergoing ART [18]. This confirms previous observations where MI was used as an adjunct before and during ovarian stimulation to ART. Wdowiak et al. suggested that MI before stimulation may reduce gonadotropin use and improve some ART outcomes [21]. The same study also reported potentially favorable findings for oocyte and embryo quality; however, these outcomes should be interpreted with caution because the evidence for them remains less consistent across the literature. In addition, Kotlyar et al., showed that patients with PCOS require careful individual tailoring of their IVF cycle to achieve optimal results. [36]. The IVM protocol can be an alternative for infertile women with PCOS who wish to prevent the potential adverse effects of gonadotropin treatment. [37]. Evidence regarding the impact of MI on oocyte and embryo quality remains limited and heterogeneous, with several studies reporting no statistically significant improvements. However, these findings should be interpreted cautiously, as the evidence regarding direct improvement in oocyte and embryo quality remains heterogeneous and not uniformly supported across studies.

### 3.3. Improving the Quality of Oocytes and Embryos

Oocyte quality is a critical factor determining the success of fertilization and embryonic development. Current findings on the influence of MI in this area are inconsistent, and therefore no firm conclusions can be drawn. Higher concentrations of MI and E2 in human FF appear to play a role in follicular maturity and provide a marker of good quality oocytes [38]. MI, through its role in insulin signaling pathways and modulation of glucose metabolism in eggs, can improve oocyte quality. Although Gupta D. et al. in their review noted that many studies indicate a tendency to improve embryo quality in women with PCOS after MI supplementation, while also highlighting the lack of statistically significant evidence that MI unequivocally improves oocyte and/or embryo quality. Clear evidence on this issue has been found to be limited [28]. Menstrual irregularity in PCOS is associated with a less favorable embryonic development profile [39]. This points to the need for further, more precisely designed research to unequivocally confirm this particular aspect. However, it should be noted that other studies, such as those cited in the ART section, indirectly indicate an improvement in oocyte quality, as better ART scores (such as increased clinical pregnancy rates) often correlate with higher oocyte and embryo quality. MI supplementation improves oocyte quality by reducing the number of degenerated and immature oocytes, in this way increasing the quality of embryos produced [40]. Current findings on this topic are inconsistent, and therefore no firm conclusions can be drawn. Although several publications have suggested a possible favorable effect of MI supplementation on oocyte and embryo quality, the available evidence remains limited and inconsistent. As noted by Gupta et al., some studies indicate a trend toward improved embryo quality in women with PCOS after MI supplementation; however, statistically conclusive evidence demonstrates a clear benefit for oocyte and/or that embryo quality is still lacking. Therefore, these outcomes should be interpreted more cautiously than ovulatory or clinical pregnancy outcomes. Overall, while some studies suggest potential benefits, the lack of consistent statistically significant results highlights the need for cautious interpretation and further well-designed clinical trials.

### 3.4. Optimizing Ovarian Response to Stimulation and Reducing Risk OHS S

Women with PCOS are often more likely to develop OHSS during ART procedures due to their numerous antral follicles and increased sensitivity to gonadotropins [41]. In this context, MI supplementation has been investigated as a supportive intervention that may help optimize ovarian responses to controlled stimulation and potentially reduce the risk of OHSS. Research suggests that MI therapy in women with PCOS results in better fertilization rates and a clear trend to a better embryo quality [42]. In a study by Beresniak A. et al., it was concluded that ovarian stimulation of rFSH in combination with oral MI supplementation in women affected by PCOS may represent a favorable strategy in terms of cost/effectiveness compared to standard stimulation of rFSH alone. However, these findings should be interpreted as supportive rather than definitive evidence [24]. MI led to a statistically significant improvement in the hormonal and metabolic profile of PCOS patients. Moreover, it is safe and has good compliance. [43]. Overall, the available evidence suggests a possible benefit of MI for ovarian response and OHSS-related outcomes, although the results remain dependent on study design and treatment protocol.

### 3.5. Evidence Stratification by PCOS Phenotypes and Metabolic Status

The clinical efficacy of MI supplementation exhibits variations when stratified by patient metabolic profiles and specific PCOS phenotypes. Regarding insulin resistance status, benefits have been documented across diverse cohorts. Specifically, mathematical modeling and prospective clinical monitoring demonstrate that MI supplementation at the standard 40:1 ratio significantly improves metabolic and hormonal parameters in both hyperinsulinemic and normoinsulinemic patients classified as Phenotype A [23]. Furthermore, when considering population characteristics such as body mass index (BMI) and age, stratified evidence reveals that the positive impact of inositol on BMI reduction is particularly pronounced in younger adults under the age of 30 [26]. This demonstrates that while MI acts fundamentally as an insulin sensitizer, its therapeutic outcomes are modulated by the baseline phenotype, age, and metabolic state of the patient.

### 3.6. Dosage Regimens, Treatment Duration, and Safety Profile

Across the included literature, intervention characteristics varied, yet consensus trends emerge regarding clinical application. The most frequently utilized formulation is MI administered alone or in a specific 40:1 ratio with D-chiro-inositol (DCI), which reflects the physiological plasma ratio [23,27]. Standard daily doses of MI typically range from 2 g to 4 g, often combined with adjunct nutraceuticals such as alpha-lipoic acid (ALA) to enhance glycemic and lipid management [20,22], or melatonin to optimize outcomes in assisted reproductive techniques (ART) [22].

The duration of treatment represents a critical variable for therapeutic success. While short-term administration can modulate acute glycolipid profiles [25], sustained clinical benefits require extended timelines. For instance, a statistically significant increase in serum sex hormone-binding globulin (SHBG) and a concurrent reduction in hyperandrogenism are primarily observed in clinical protocols where MI is administered continuously for at least 24 weeks [29].

Regarding safety, MI supplementation demonstrates an excellent tolerability profile. In comparative clinical analyses, optimal doses of MI effectively lower triglyceride levels while successfully avoiding the gastrointestinal adverse effects commonly associated with traditional pharmacological insulin sensitizers like metformin [25]. No severe adverse events, long-term toxicities, or absolute contraindications were highlighted within the reviewed clinical protocols, positioning MI as a safe adjunct therapy. The duration of treatment represents a critical variable for therapeutic success. While short-term administration can modulate acute glycolipid profiles [25], sustained clinical benefits require extended timelines. For instance, a statistically significant increase in serum sex hormone-binding globulin (SHBG) and a concurrent reduction in hyperandrogenism are primarily observed in clinical protocols where MI is administered continuously for at least 24 weeks [29].

Regarding safety, MI supplementation demonstrates an excellent tolerability profile. In comparative clinical analyses, optimal doses of MI effectively lower triglyceride levels while successfully avoiding the gastrointestinal adverse effects commonly associated with traditional pharmacological insulin sensitizers like metformin [25]. No severe adverse events, long-term toxicities, or absolute contraindications were highlighted within the reviewed clinical protocols, positioning MI as a safe adjunct therapy (Table 3).

## 4. Limitations and Future Research

This scoping review has several limitations that should be considered when interpreting its findings. A fundamental limitation is the scoping review methodology used, which by its nature does not include a formal assessment of the methodological quality of the included studies [15]. The potential heterogeneity of the included studies is also a significant challenge and limitation. This heterogeneity is manifested in differences in supplementation protocols, where the doses of MI, DCI and their proportions are variable, as well as the combinations used with other active substances, such as ALA or melatonin [22,27]. Such diversity makes it difficult to identify a single, optimal supplementation formula. In addition, there are significant differences in the characteristics of the study population, including variables such as age, PCOS phenotype, metabolic status (e.g., hyperinsulinemia or normoinsulinemia) and diagnostic criteria used in individual cohorts [23]. There were also discrepancies in the endpoints measured, from hormonal and metabolic parameters to detailed fertility and ART-related outcomes, which makes direct comparisons difficult. The duration of MI supplementation interventions, ranging from a few weeks to many months, is another factor influencing the observed effects. All these factors may contribute to the heterogeneity of the presented results, as exemplified by the lack of statistically significant evidence for a clear improvement in the quality of oocytes and embryos, despite the tendency to improve in some studies [28]. Based on these mapped limitations, future clinical research must transition from broad, generalized efficacy evaluations to highly specific, protocol-driven investigations. Rather than executing broad-cohort trials, upcoming multicenter randomized controlled trials (RCTs) should mandate strict participant stratification by specific Rotterdam diagnostic phenotypes (Phenotypes A, B, C, and D) and baseline metabolic status (hyperinsulinemic vs. normoinsulinemic) to precisely identify which patient subgroups derive the greatest clinical benefit. Furthermore, future study designs must standardize supplementation protocols by establishing direct, head-to-head pharmacological comparisons between MI monotherapy and fixed-ratio formulations (such as the 40:1 MI:DCI ratio), while defining uniform intervention timelines restricted to rigid periods (minimum 24 weeks) to adequately evaluate long-term metabolic shifts. Finally, future reproductive outcomes must prioritize definitive clinical endpoints, such as live birth rates and cumulative pregnancy rates, alongside comprehensive tracking of long-term safety profiles and adverse events, rather than relying predominantly on proxy surrogate markers like oocyte morphology or transient hormonal fluctuations. Addressing these explicit methodological gaps is a prerequisite for translating scoping data into robust, personalized clinical guidelines.

## 5. Conclusions

As a scoping review, this work aims to map and summarize the available evidence rather than to draw causal or definitive conclusions regarding the effectiveness of MI supplementation. The findings presented here reflect the scope and characteristics of the existing literature and should not be interpreted as clinical recommendations or confirmatory evidence. Given the heterogeneity of study designs, populations and outcomes, the available evidence remains insufficient to support firm conclusions, and the results should be interpreted with appropriate caution. This review does not attempt to evaluate the efficacy of MI but instead provides an overview of how its effects have been reported across different studies. The purpose of this review is descriptive rather than evaluative, and the synthesis presented here reflects the diversity, gaps and trends in the current evidence base.

This scoping review, based on a comprehensive analysis of the available literature, suggests that MI supplementation has a clear and multidirectional positive effect on fertility and ovarian function in women with PCOS. The available studies indicate that MI may contribute to the improvement of ovulatory function, restoration of menstrual cycle regularity, ovulatory function, and increased chances of conception. MI supplementation, often in combination with DCI, has also been associated with improved clinical pregnancy rates, both in spontaneous pregnancies and those obtained in the context of ART. Although some analyses did not demonstrate statistically significant evidence for a clear improvement in the quality of oocytes and embryos, many studies indicate a positive trend in this area. In addition, MI supplementation may be beneficial in reducing the risk of OHSS, optimizing ovarian response to stimulation and increasing the safety of ART procedures. However, the available evidence remains heterogeneous in terms of supplementation protocols, study populations, and reported outcomes. Therefore, although MI appears to be a promising supportive intervention in women with PCOS, further well-designed studies are needed to clarify its effects across specific reproductive and metabolic outcomes.

## Figures and Tables

**Table 1:** Summary of search strategy.

Database	Date of Search	Full Search String/Controlled Vocabulary	Filters Applied
**PubMed**	10 January 2026	(“Myo-inositol” [MeSH] OR “myo-inositol” OR myoinositol OR inositol OR “D-chiro-inositol” OR Dchiroinositol OR “inositol supplementation”) AND (“Polycystic Ovary Syndrome” [MeSH] OR PCOS OR “polycystic ovarian syndrome” OR “Stein-Leventhal syndrome”)	English; Humans; Adults (≥18 years); Full text; Article types: Clinical Study, Observational Study, RCT, Review, Systematic Review, Meta-analysis
**Scopus**	12 January 2026	TITLE-ABS-KEY (“myo-inositol” OR myoinositol OR inositol OR “D-chiro-inositol” OR Dchiroinositol OR “inositol supplementation”) AND TITLE-ABS-KEY (PCOS OR “polycystic ovarian syndrome” OR “Stein-Leventhal syndrome”)	English; Article; Review; Adult
**Web of Science (Core Collection)**	14 January 2026	TS = (“myo-inositol” OR myoinositol OR inositol OR “D-chiro-inositol” OR Dchiroinositol OR “inositol supplementation”) AND TS = (PCOS OR “polycystic ovarian syndrome” OR “Stein-Leventhal syndrome”)	English; Document types: Article, Review; Adult
**EBSCO—MEDLINE Complete**	15 January 2026	(MH “Inositol” OR “myo-inositol” OR myoinositol OR inositol OR “D-chiro-inositol” OR Dchiroinositol) AND (MH “Polycystic Ovary Syndrome” OR PCOS OR “polycystic ovarian syndrome” OR “Stein-Leventhal syndrome”)	English; Peer-reviewed; Full text; Adults
**Cochrane Library**	17 January 2026	(“myo-inositol” OR myoinositol OR inositol OR “D-chiro-inositol” OR Dchiroinositol) AND (PCOS OR “polycystic ovarian syndrome” OR “Stein-Leventhal syndrome”)	English; Trials; Reviews

**Table 2:** Characteristics and findings of studies included in this review.

Author, Year	Country	Aim of the Study	Participants	Type of Research	Results and Findings	Effects of Treatment (P/N/U)
Sene AA. et al., 2025 [18]	Iran	evaluate the effect of MI and DCI on ART outcomes in women with PCOS.	N/A	Review	- Reliable evidence has been provided for the effects of MI/DCI on fertility and ovarian function in women with PCOS undergoing ART - MI/DCI supplementation significantly increased clinical pregnancy rates	P
van der Wel AWT. et al., 2025 [19]	The Netherlands	To evaluate the effect of daily MI supplementation during pregnancy in patients with PCOS on the risk of diabetes, preeclampsia and preterm birth.	464 participants	Randomized Controlled Trial	- MI supplementation during pregnancy did not reduce the incidence of gestational diabetes, preeclampsia, or preterm birth in patients with PCOS	N
Firat S. et al., 2025 [20]	Turkey	evaluate the impact of MI and ALA supplementation on hormonal and metabolic markers in women diagnosed with PCOS.	58 women	retrospective case–control study	- Adding MI + ALA supplementation to standard PCOS treatment may offer metabolic benefits, particularly in maintaining glycemic control, body weight, and BMI - Supplementation also reduces low-density lipoprotein (LDL)	P
Wdowiak A. et al., 2025 [21]	Poland	Evaluation of the use of MI in the procedure supporting reproductive techniques.	N/A	Narrative Review	- The research discussed in this article shows the great potential of MI in both PCOS and non-PCOS patients seeking in vitro fertilization (IVF) care - The use of MI before ovarian stimulation may positively affect the use and duration of gonadotropin, oocyte and embryo quality, fertilization, and clinical pregnancy rates	P/U
Saxena A. et al., 2024 [22]	India	evaluated the efficacy of optimal nutraceutical combinations in improving PCOS characteristics using system biology-based mathematical modelling and simulation.	2000 woman with PCOS	Study in silico	- Supplementation with a combination of MI, melatonin, and ALA has shown potential in the treatment of PCOS symptoms	P
Pustotina O. et al., 2024 [23]	Russia	evaluates the frequency of metabolic abnormalities in PCOS patients and the effects of MI and DCI, in a 40:1 ratio on hormonal and metabolic parameters.	40 patients with PCOS	Prospective study	- MI supplementation improved metabolic and hormonal profile - Positive results were observed in both patients with hyperinsulinelinemia and normoinsulinemic	P
Beresniak A. et al., 2023 [24]	Italy	to evaluate the outcomes and the costs of IVF cycles performed in PCOS individuals, where the stimulation with rFSH is associated with MI oral supplementation compared with standard stimulation protocols with Recombinant Follicle-Stimulating Hormone (rFSH) only.	N/A	Computer simulation/economic modeling	- Study suggests potential benefits of oral MI supplementation during ART procedures in terms of cost/effectiveness - Ovarian stimulation of rFSH and MI in women affected by PCOS can be considered the dominant strategy compared to standard stimulation of rFSH alone	P
Zhang JQ. et al., 2022 [25]	China	perform an updated meta-analysis to evaluate MI and the classical insulin sensitizer metformin in terms of efficacy and safety for treating women with PCOS.	612 patients	Meta-analyze	- Compared to metformin, an appropriate MI supplementation dose may be helpful in lowering TG levels and avoiding side effects	P
Zarezadeh M. et al., 2021 [26]	Iran	assess the impact of inositol supplementation on BMI through a systematic review and meta-analysis of controlled clinical trials.	N/A	Review	- Positive effects of inositol on BMI in adults under 30 years of age, as well as in people with PCOS - Inositol supplementation can be administered as an adjunct therapy to improve anthropometric indicators and glycemic responses	P
Dinicola S. et al., 2021 [27]	Italy	review of current knowledge about inositols (MI and DCI).	N/A	Review	- Despite the evidence, the validity of the MI to DCI 40:1 ratio should be further elucidated and supported by large-scale clinical trials as well as pharmacokinetic studies - In PCOS, there is an “ovarian paradox” - MI is effective and safe for PCOS	P
Gupta D. et al., 2020 [28]	UK	review and compare the existing studies and literature to assess the impact of MI on oocyte and embryo quality in ARTs.	N/A	Review	- Many of the studies carried out have shown a tendency to improve embryo quality in women with PCOS after supplementation; however, there is a lack of statistically significant evidence to support the use of MI in improving oocyte and/or embryo quality - Clear evidence for the role of MI in improving oocyte and embryo quality in PCOS is limited	U
Unfer V. et al., 2017 [29]	Switzerland	assess the effects of MI alone or combined with DCI on the endocrine and metabolic abnormalities of women with PCOS.	N/A	Meta-Analyze	- There was a slight trend towards a decrease in testosterone levels by MI - A significant increase in serum SHBG was observed only in those studies in which MI was administered for at least 24 weeks - These results highlight the beneficial effect of MI in improving the metabolic profile of women with PCOS while reducing their hyperandrogenism	P
Rolland AL. et al., 2017 [30]	France	study whether supplementation with MI can improve patients’ sensitivity to clomiphene citrate (CC) in terms of ovulation and pregnancy rates.	26 patients with PCOS	Pilot study	- A pilot study appeared to demonstrate the benefits of MI supplementation during ovulation induction from CC in patients with PCOS	P

P—positive; N—negative; U—unknown.

**Table 3:** Summary of the most important aspects of the impact of MI supplementation on fertility and ovarian function in PCOS.

Impact Aspect	Description of the Effect of MI	Key Mechanisms and Evidence
Improving ovulatory function	MI has been associated with improved menstrual cycles regularity and ovulatory induction in women with PCOS, who often suffer from anovulation or oligoovulation. This appears to be one of the more consistently reported reproductive outcomes of supplementation [5,7,9].	As a secondary transmitter of the insulin signal, MI improves the sensitivity of ovarian cells to insulin. It reduces insulin resistance and hyperinsulinemia, which leads to a decrease in the level of ovarian androgens, which block follicle development and ovulation [3,5,6]. Meta-analyses consistently show an increase in ovulation rate, although study heterogeneity should be taken into account [7].
Increase pregnancy rates	MI supplementation, especially in combination with DCI, has been associated with improved clinical pregnancy rate, both in the case of spontaneous and ART [10,11]. This is observed in various infertility treatment strategies.	Improving ovulatory function and cycle regularity may contribute to better chances of spontaneous pregnancy. In ART, MI contributes to better ovarian preparation, which indirectly affects higher fertilization and implantation rates [10].
Improve results ART	The use of MI before and during ART, such as IVF, improves their effectiveness. It has a positive effect on gonadotropin consumption (the possibility of using lower doses or shorter time), the quality of oocytes and embryos, which leads to higher fertilization rates and clinical pregnancies [11,12]. However, these findings are not uniform across all studies. MI supplementation has also been described as potentially favorable from cost/effectiveness compared to standard stimulation protocols [19].	MI may optimize the microenvironment of the ovarian follicle, which affects the maturation of oocytes and their developmental competence [16]. Improved ovarian responsiveness to stimulation by exogenous hormones may contribute to a higher proportion of mature oocytes and embryos with favorable morphology, which could positively influence ART success rates [12,13].
Optimization of oocyte quality	MI has the potential to improve the quality of oocytes, which is crucial for proper fertilization and embryo development. Although the evidence is mixed and requires further investigation, many analyses point to a trend towards better oocyte and embryo quality after MI supplementation [13,15].	MI, by influencing insulin signaling pathways and glucose metabolism in egg cells, supports the normal growth and maturation of oocytes. It can reduce the number of immature oocytes and increase the number of those that have undergone meiosis and are ready for fertilization [12].
Risk reduction OHSS	MI supplementation may help optimize ovarian responses to controlled gonadotropin stimulation while reducing the risk of OHSS, which is a common complication in women with PCOS undergoing ART [17,18,19].	MI can increase the sensitivity of the ovaries to gonadotropins, which allows for the use of lower doses of stimulants, thus minimizing the risk of overreaction. It can also improve the synchronization of follicle maturation, reducing the number of follicles that become prone to hyperstimulation [18,20].
Modulation of hormonal and metabolic parameters	MI contributes to the improvement of hormonal (e.g., lowering androgen levels) and metabolic profile (improving insulin sensitivity, glycemic control) [7,12,13]. Although this is a broader effect of MI, it has a direct impact on ovarian health and the ability to ovulate properly.	Reduction in insulin resistance and hyperinsulinemia, which are pathogenically associated with ovarian dysfunction and hyperandrogenism in PCOS. Improving these parameters creates a more conducive environment for healthy ovarian follicle development and the restoration of hormonal balance [3,4,6].
Clinical Practical Points	Clear guidelines on dosage, treatment duration and patient selection optimize the real-world clinical utility of MI.	Standard recommendation involves 2–4 g/day of MI ideally in a 40:1 MI:DCI ratio [23,27]. Treatment must be maintained for a minimum of 24 weeks to achieve significant endocrinological changes (e.g., SHBG elevation) [29]. It serves as a highly tolerable, low risk alternative for patients prone to metformin-induced adverse effects [25].

## Data Availability

No new data were created or analyzed in this study.

## Supplementary Materials

The following supporting information can be downloaded at: https://www.mdpi.com/article/10.3390/nu18132093/s1, File S1: Preferred Reporting Items for Systematic reviews and Meta-Analyses extension for Scoping Reviews (PRISMA-ScR) Checklist [16].

## Abbreviations

PCOS	Polycystic ovary syndrome
MI	Myo-inositol
JBI	Joanna Briggs Institute
PCC	Population-Concept-Context
ART	Assisted reproductive techniques
OHSS	Ovarian hyperstimulation syndrome
PRISMA-ScR	Preferred Reporting Items for Systematic Reviews and Meta-Analyses for Scoping Reviews
MeSH	Medical Subject Headings
ALA	α-lipoic acid
BMI	Body Mass Index
LDL	Low-density lipoprotein
rFSH	Recombinant Follicle-Stimulating Hormone
DCI	d-chiro-inositol
CC	clomiphene citrate
IVF	in vitro fertilization
RCTs	randomised controlled trials
